# A Conditioning-Strengthened Circuit From CA1 of Dorsal Hippocampus to Basolateral Amygdala Participates in Morphine-Withdrawal Memory Retrieval

**DOI:** 10.3389/fnins.2020.00646

**Published:** 2020-07-14

**Authors:** Qianqian Ma, Yali Fu, Zixuan Cao, Da Shao, Jiaojiao Song, Huan Sheng, Li Yang, Dongyang Cui, Ming Chen, Fei Zhao, Min-Hua Luo, Bin Lai, Ping Zheng

**Affiliations:** ^1^State Key Laboratory of Medical Neurobiology, Department of Neurology of Zhongshan Hospital, MOE Frontier Center for Brain Science, School of Basic Medical Sciences, Institutes of Brain Science, Fudan University, Shanghai, China; ^2^School of Basic Medical Sciences, Capital Medical University, Beijing, China; ^3^Chinese Institute for Brain Research, Beijing, China; ^4^State Key Laboratory of Virology, CAS Center for Excellence in Brain Science and Intelligence Technology, Center for Biosafety Mega-Science, Wuhan Institute of Virology, Chinese Academy of Sciences, Wuhan, China; ^5^University of Chinese Academy of Sciences, Beijing, China

**Keywords:** addiction, morphine, basolateral amygdala, CA1 of dorsal hippocampus, postrhinal cortex, memory retrieval

## Abstract

Conditioned context-induced retrieval of drug withdrawal memory contributes to drug relapse. The basolateral amygdala (BLA) is an important brain region that is involved in conditioned context-induced retrieval of morphine withdrawal memory. However, the upstream pathways of the activation of the BLA by conditioned context remains to be studied. The present results show that the CA1 of dorsal hippocampus is an upstream brain region of the activation of the BLA during conditioned context-induced morphine withdrawal memory retrieval; the indirect connection from the CA1 of dorsal hippocampus to the BLA is enhanced in mice with conditioned place aversion (CPA); the postrhinal cortex (POR) is a brain region that connects the CA1 of dorsal hippocampus and the activation of the BLA during conditioned context-induced retrieval of morphine-withdrawal memory. These results suggest that a conditioning-strengthened indirect circuit from the CA1 of dorsal hippocampus to the BLA through the POR participates in morphine withdrawal memory retrieval.

## Introduction

Drug addiction is a chronic brain disorder characterized by compulsive drug seeking and use (Borelli, 2009). The most insidious feature of drug addiction is a high rate of drug relapse. One important factor that contributes to the relapse is conditioned context-induced retrieval of drug withdrawal memory. Mainly, drug withdrawal is associated with environmental context through a process termed “conditioning,” the previous neutral context acquires the capability to trigger the retrieval of drug withdrawal memory, leading to drug relapse (Crombag et al., 2008).

Previous studies found that the basolateral amygdala (BLA) played an important role in the conditioned context-induced retrieval of morphine withdrawal memory (Lovaglio et al., 2010; Garcia-Perez et al., 2016; Valero et al., 2018; Song et al., 2019). However, how conditioned context activates the BLA to participate in conditioned context-induced retrieval of morphine withdrawal memory remains to be unknown.

We proposed that the hippocampus might be an important upstream brain region of the activation of the BLA during conditioned context-induced retrieval of morphine withdrawal memory because the hippocampus was critical for learning about context (Arias et al., 2015) and could encode the association of the context with the related response such as shock (Arias et al., 2015) and reward (Hearing et al., 2010). To test this hypothesis, we studied the role of the hippocampus, and examined the relationship of the hippocampus with the activation of the BLA during conditioned context-induced retrieval of morphine withdrawal memory.

The hippocampus has different portions along its dorsal-ventral axis having different roles, due to differences in connectivity (Daumas et al., 2005). Previous studies showed that both the dorsal and ventral hippocampus were important for conditioned context-induced retrieval of fear memory (Xu et al., 2016). However, the role of the dorsal and ventral hippocampus in conditioned context-induced retrieval of drug withdrawal memory remains to be unknown. Here, we used the immunohistochemical method to examine the influence of conditioned context on the expression of c-Fos, a marker of neuronal activity (Dragunow and Faull, 1989), in the CA1 of dorsal and ventral hippocampus, and then studied the role of this activation in conditioned context-induced retrieval of morphine withdrawal memory by examining the influence of the inactivation of these regions by the local injection of GABA_A_ receptor agonist muscimol on conditioned place aversion (CPA) in morphine withdrawn mice. Our results showed that only the dorsal hippocampus played an important role in conditioned context-induced retrieval of morphine withdrawal memory, and the inhibition of the CA1 of dorsal hippocampus could significantly inhibit the conditioned context-induced activation of the BLA in morphine withdrawn mice. However, up to now, no reports show that the dorsal hippocampus has direct projections to the BLA. Therefore, it is possible that the CA1 of dorsal hippocampus activates the BLA by an indirect pathway during conditioned context-induced retrieval of morphine withdrawal memory. To test this hypothesis, we used neural circuit tracing technique to study the connection from the CA1 of dorsal hippocampus to the BLA and examined whether there was a change in the connection in mice with CPA. In addition, we further studied the brain regions that connected the CA1 of dorsal hippocampus and the activation of the BLA during conditioned context-induced retrieval of morphine withdrawal memory using the transneuronal virus tracing technique combined with the chemical-genetic method.

## Materials and Methods

### Animals

Male adult (6–8 weeks) C57BL/6J mice were housed singly in a 12 h light/dark cycle (lights on 7:00 AM to 7:00 PM) in a temperature and humidity controlled environment with food and water freely available. All experimental procedures conformed to the Fudan University as well as international guidelines on the ethical use of animals. All efforts were made to minimize animal suffering and reduce the number of animals used.

### Cannula Placement

Mice were anesthetized with ketamine and xylazine (160 mg/kg and 12 mg/kg body weight, respectively) and placed in the stereotaxic instruments (RWD, Shenzhen, China). For microinjection, injection needles were connected to a 1 μl microsyringe (Hamilton) by polyethylene tubing and controlled by a syringe pump (Harvard Apparatus). Mice were implanted with two stainless-steel guide cannulas (cannulae for the CA1 of dorsal hippocampus: O.D. 0.48 mm × I.D. 0.34 mm, C = 1.0 mm; cannulae for the POR: O.D. 0.41 mm × I.D. 0.25 mm; C = 2.3 mm) bilaterally 1 mm above each side of the CA1 of dorsal hippocampus (AP,-2.0; ML, ± 1.5; DV, −1.6) and the POR (AP, −4.28; ML, ± 4.1; DV, −3.1), based on the atlas of Franklin et al. (2008). Two anchoring screws and dental cement secured the cannulae to the skull. Stainless steel stylets were inserted into the cannulae to prevent occlusion. After surgery, animals were housed individually and were allowed to recover for more than 1 week before performing procedure for CPA testing. Mice were bilaterally injected with muscimol (0.11 mg/ml in 0.9% saline, 0.3 μl each side) for 1 min at 30 min before post-conditioning test. After the injection, the needles were retained in place for another 1 min. The number of excluded missed-site stereotaxic placement animals did not exceed 10% of the sample.

### Biotinylated Dextran Amine Injection

After the mice were anesthetized with ketamine and xylazine (160 mg/kg and 12 mg/kg body weight, respectively) and secured in the stereotaxic instruments (RWD, Shenzhen, China), anterograde tracing marker, biotinylated dextran amine (BDA) (10,000 MW, D-1956; Invitrogen; 10% dissolved in 0.1 M PH 7.4 phosphate buffer), was injected into the CA1 of dorsal hippocampus and the CA1 of ventral hippocampus (AP, −3.16; ML, ±3.8; DV, −4.1) based on the atlas of Franklin et al. (2008) in a volume of 0.3 μl and retained in place for an additional 10 min to optimize diffusion. Following the BDA tracing in axons for 7–10 days, mice were perfused with 0.9% saline and followed by 4% paraformaldehyde in phosphate buffer (PFA, pH 7.4). Then, the brains were removed and post-fixed in 4% PFA for 18–24 h. All brains were cut in 40 μm coronal sections on a vibration microtome (Leica) and collected in 0.01 M PBS. The mice with a wrong injection site were not included in data analysis.

### Chronic Morphine Treatment

Male adult C57BL/6J mice were treated with morphine according to procedures described previously (Vindenes et al., 2006). Briefly, mice were injected with daily escalating doses of morphine (10 mg/kg on day 1, 20 mg/kg on day 2, 30 mg/kg on day 3, 40 mg/kg on day 4, and day 5, i.p.) twice a day at 8:00 AM and 7:00 PM. Mice in control groups were treated with equivalent volume of saline following the same procedure.

### Conditioned Place Aversion

Conditioned place aversion was conducted with a three-compartment place conditioning apparatus (Med Associates, United States) with distinct visual and tactile environments to maximize contextual differences. The procedure for CPA was similar to that described previously (Chen et al., 2019). On day 1, the pre-conditioning phase (pre-test), the animals were placed in the central neutral area of the apparatus for 2 min to further habituation and then allowed to freely explore all three compartments for 15 min. Animals showing a strong preference (>80% of the session time) or aversion (<20% of the session time) for any compartment were discarded from the study, the number of excluded animals does not exceed 10% of the sample. All animals having no unbiased nature for any compartment after the criteria of assessment were randomly divided into four groups: saline + saline, saline + naloxone, morphine + saline, and morphine + naloxone. On day 2–6, these animals received daily escalating doses of morphine (i.p.) or saline (i.p.) as described above. Naloxone is an opioid receptor antagonist and can terminate chronic morphine exposure by displacing morphine from the opioid receptors for its higher affinity with Mu receptors than that of morphine, thus precipitating withdrawal syndromes. On day 7 and day 9, 2 h after treatment with morphine or saline, the animals received a subcutaneous injection of naloxone (0.3 mg/kg) or saline and were immediately restricted to one compartment for 20 min. On day 8 and day 10, 2 h after morphine or saline treatment, they received a subcutaneous injection of saline and were immediately restricted to the opposite compartment for 20 min. On day 11, 24 h after the last conditioning session, animals were re-exposed to the CPA compartments and were allowed to freely explore the entire apparatus for 15 min. CPA score was defined as difference between the time spent in the saline-paired compartment and the time spent in the naloxone-paired compartment (the time in the naloxone-paired compartment minus the time in the saline-paired compartment) (Wang et al., 2017; Chen et al., 2019).

### Virus Projection

Eight-week-old C57/BL6 male mice were anesthetized with ketamine and xylazine (160 and 12 mg/kg body weight, respectively), and placed in stereotaxic instruments (RWD, Shenzhen, China). For the inhibitory experiment of CA1 of dorsal hippocampus-POR projection, each side of the CA1 of dorsal hippocampus was injected with 0.3 μl AAV-hSyn-DIO-hM4D (Gi)-EGFP (3.44 × 10^12^ vector genomes/ml, GeneChem Company, Shanghai, China) for 10 min followed by an additional 10 min to allow the diffusion of virus. Each side of the POR was injected with 0.3 μl AAV-hSyn-mCherry-IRES-WGA-Cre (4.28 × 10^12^ vector genomes/ml, GeneChem Company, Shanghai, China). For the inhibitory experiment of POR-BLA projection, each side of POR was injected with 0.3 μl AAV-hSyn-DIO-hM4D(Gi)-EGFP (3.44 × 10^12^ vector genomes/ml, GeneChem Company, Shanghai, China) for 10 min followed by an additional 10 min to allow the diffusion of virus. Each side of the BLA was injected with 0.3 μl AAV-hSyn-mCherry-IRES-WGA-Cre (4.28 × 10^12^ vector genomes/ml, GeneChem Company, Shanghai, China). The viruses were allowed to express for at least 4 weeks in order to allow sufficient accumulation. The mice were treated with saline or CNO (10 mg/kg, i.p. MedChemExpress (MCE) company, Cat# HY-17366) 45 min before post-test.

H129-G4 virus (2.45 × 10^12^ vector genomes/ml, granted from Wuhan Institute of Virology, China) was injected into the CA1 of dorsal hippocampus in a volume 120 nl and retained in place for 10 min to optimize diffusion. 36 h after the H129-G4 injection, mice were perfused with 0.9% saline and followed by 4% PFA. Then, the brains were removed and post-fixed in 4% PFA at 4°C for 18–24 h. All brains were cut in 40 μm coronal sections on a vibration microtome (Leica) and collected in 0.01 M PBS. The mice with a wrong injection site were not included in data analysis.

### Two-Step Virus Injection Approach

A total of 300 nl pAAV-hSyn-Cre-EGFP (1 × 10^13^ vector genomes/ml, GeneChem Company, Shanghai, China) was injected into each side of the CA1 of dorsal hippocampus to label the POR neurons receive the input from CA1 of dorsal hippocampus. It can directly infect the postsynaptic neurons in a target region that specifically receive input from CA1 of dorsal hippocampus and enable Cre-dependent transgene expression in nucleus. If the POR neurons receive the input from CA1 of dorsal hippocampus, they will be labeled with green fluorescence (EGFP) and express Cre protein. One week later, 300 nl pAAV-pCAG-FLEX-tdTomato-WPRE (1.2 × 10^13^ vector genomes/ml, GeneChem Company, Shanghai, China) was injected into each side of the POR to detect the project regions of those EGFP positive POR neurons receiving input from CA1 of dorsal hippocampus. In the POR, only those neurons receiving input from CA1 of dorsal hippocampus can express the tdTomato by a Cre-dependent method. The tdTomato protein will also be expressed in the axon terminal and can be detected in the project regions. So by detecting the fluorescence of tdTomato in the BLA, we can estimate whether those EGFP positive POR neurons receiving input from CA1 of dorsal hippocampus project to BLA region.

### Immunofluorescent Staining

Male adult C57BL/6J mice were anesthetized with ketamine and xylazine (160 mg/kg and 12 mg/kg body weight, respectively) 90 min after the end of CPA testing and perfused with 0.9% saline followed by ice-cold solution of 4% PFA. Their brains were removed and fixed in 4% PFA at 4°C for 18–24 h and then were cut into 40 μm coronal sections using a vibratome (VT-1000S, Leica, Germany). The brain slices of CA1 of dorsal hippocampus (range relative to bregma: −1.58 ∼−2.18 mm), CA1 of ventral hippocampus (range relative to bregma: −2.92 ∼−3.64 mm), BLA (range relative to bregma: −1.24 ∼−1.60 mm), LS (lateral septal nucleus) (range relative to bregma: +0.86 ∼ +0.14 mm) and POR (range relative to bregma: −4.10 ∼−4.48 mm) were collected in 0.01 M PBS. Free-floating sections containing these regions were rinsed in PBS three times. Subsequently, sections were incubated with blocking solution containing 10% normal goat serum and 0.3% Triton X-100 in PBS for 2 h at 37°C. Sections were then incubated with rabbit anti-c-Fos antibody (1:500, Synaptic Systems, Germany, Cat#226003) overnight at 4°C. Subsequently, they were washed with PBS three times and incubated with Alexa Fluor 594-conjugated goat anti-rabbit antibody (1:200, Abcam, Cat#150088) for 1 h at 37°C. Finally, immunolabeled sections were rinsed three times with PBS, and mounted on glass slides using aqua-mount mounting medium. All antibodies were dissolved in PBS with 10% normal goat serum and 0.3% Triton X-100. A series of slices containing these regions were imaged by confocal microscopy (Nikon AIR-MP) with a 20 × −immersion lens and collected at a resolution of 1024 × 1024 pixels. The same laser and scanning settings were used for all confocal images within an experiment to allow for comparison across groups. Generally, coronal sections from 5 to 7 animals were used for quantitative analysis and 6–8 images of each slice were averaged to determine a value for the slice. Series of images were captured from the confocal microscope and converted to 8-bit gray scale images. Then the area of white color clusters was measured using the Image-Pro-Plus 6.0 software. Quantification of c-Fos labeled neurons was estimated in the form of optical density with the same threshold. The positive cells were defined with large nuclei stained diffusely and staining above basal background. Before cell counting, the boundary of these regions was drawn on the picture based on the atlas of Franklin et al. (2008) and only the positive cells within the boundary were counted.

### Drugs and Materials

Morphine was purchased from Shenyang No.1 Pharmaceutical Factory, China. 0.01 M PBS, Triton X-100, naloxone and muscimol were purchased from Sigma, United States. Rabbit anti-c-Fos antibody (Cat#5348) was purchased from Cell Signaling Technology, United States. Goat serum and Alexa Fluor 594-conjugated goat anti-rabbit antibody were purchased from Jackson Immuno Research Laboratory, United States. Biotinylated anti-rabbit secondary antibody was purchased from Vector Laboratories, United States. Other reagents in artificial cerebrospinal fluid (ACSF) were the products of Shanghai Chemical Plant, China.

### Off-Line Data Analysis

Off-line data analysis was performed with SigmaPlot (Jandel Scientific) and Image-Pro-Plus 6.0. In all cases, *n* refers to the number of animals. Statistical significance was determined using Student’s *t*-test for comparisons between two groups. For multi-groups of samples, the statistical significance was analyzed with one-way ANOVA following by Tukey *post hoc* analysis. Two-way ANOVA with repeated measures was used to evaluate the influences of the two within-subject factors and Bonferroni *post hoc* test was used to detect the subgroup differences after the ANOVA comparison. For all results, *p* < 0.05 was accepted as being statistically significant.

## Results

### CA1 of Dorsal Hippocampus Plays an Important Role in Conditioned Context-Induced Retrieval of Morphine-Withdrawal Memory, but CA1 of Ventral Hippocampus Does Not

**FIGURE 1:**
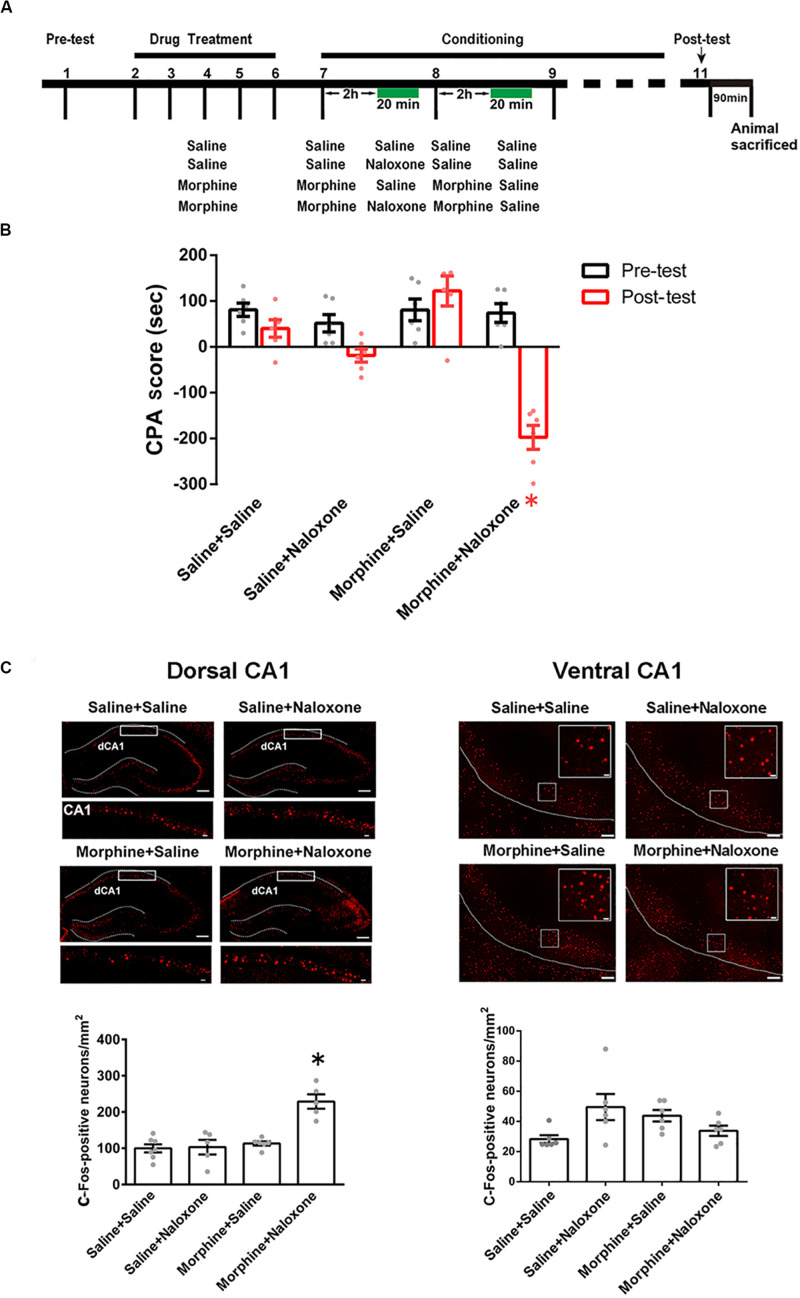
The influence of conditioned context on c-Fos expression in the CA1 of dorsal and ventral hippocampus in morphine withdrawn mice. **(A)** The experimental timeline and groups for the CPA procedure. **(B)** The CPA score of each group (*n* = 6 in each group, *p = 0.0037, compared with pre-test, two-way ANOVA, Bonferroni *post hoc* analysis). **(C)** Left top panel: C-Fos positive neurons (red-colored) of the CA1 of dorsal hippocampus in each group. Scale bar = 100 μm. Higher magnification images of boxed regions are shown on the bottom. Scale bar = 20 μm. Left down panel: average number of c-Fos positive neurons in the CA1 of dorsal hippocampus of each group (*n* = 6 in saline + saline group and morphine + saline group, *n* = 5 in saline + naloxone group and morphine + naloxone group, *p < 0.0001, one-way ANOVA following by Tukey *post hoc* analysis). Right top panel: C-Fos positive neurons (red-colored) of the CA1 of ventral hippocampus in each group. Scale bar = 100 μm. Higher magnification images of boxed regions are shown on the right. Scale bar = 20 μm. Right down panel: average number of c-Fos positive neurons of the CA1 of ventral hippocampus of different groups (*n* = 6 in each group). Data are shown as the mean ± SEM.

To study the role of the CA1 of dorsal and ventral hippocampus in conditioned context-induced retrieval of morphine withdrawal memory, we studied whether conditioned context could activate the CA1 of dorsal and ventral hippocampus by examining the expression of c-Fos, a molecular marker of neuronal activation (Joo et al., 2016), in the CA1 of dorsal and ventral hippocampus in morphine withdrawn mice. Mice were randomly divided into four groups: saline + saline, saline + naloxone, morphine + saline, and morphine + naloxone, as described in the method section and were subjected to behavioral procedure as shown in Figure 1A. The results showed that the mice in morphine + naloxone group exhibited a strong aversion to withdrawal-paired compartment and thus spent less time in the withdrawal-paired compartment during the post-test than that during the pre-test, producing an increase in “aversion score” (CPA score) (drug factor, *F*_(3, 40)_ = 20.27, *p* < 0.0001; test factor, *F*_(1, 40)_ = 30.27, *p* < 0.0001; drug × test, *F*_(3, 40)_ = 18.30, *p* < 0.0001; two-way ANOVA, Bonferroni *post hoc* analysis, Figure 1B), whereas mice in other groups did not exhibit a significant aversion to either compartment. On this basis, we examined the expression of c-Fos in the CA1 of dorsal and ventral hippocampus at 90 min after post-test. Upper panels of Figure 1C showed confocal images of c-Fos positive neurons and lower panels of Figure 1C were the average numbers of c-Fos positive neurons in the CA1 of dorsal and ventral hippocampus in each group. We could see that the expression of c-Fos in the CA1 of dorsal hippocampus significantly increased in the morphine + naloxone group after the re-exposure to conditioned context (*F*_(3, 19)_ = 17.21, *p* < 0.0001; one-way ANOVA followed by Tukey’s multiple comparison test, left down panel of Figure 1C), but did not in the CA1 of ventral hippocampus (*F*_(3, 20)_ = 3.424, *p* > 0.05; one-way ANOVA followed by Tukey’s multiple comparison test, right down panel of Figure 1C). This result suggests that conditioned context re-exposure can activate CA1 neurons of the dorsal hippocampus, but does not activate CA1 neurons in the ventral hippocampus in morphine withdrawn mice.

**FIGURE 2:**
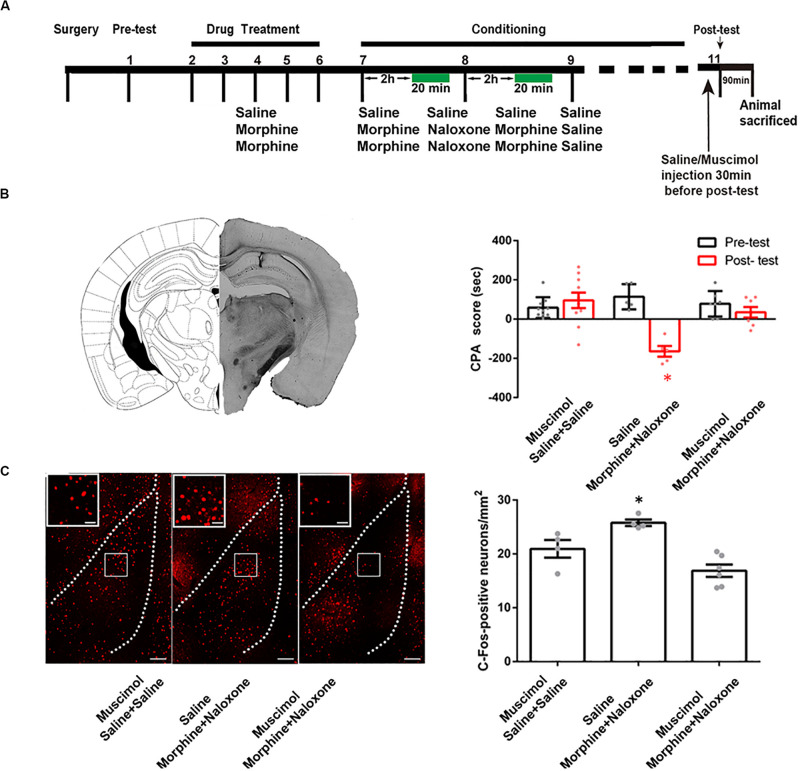
The influence of the inactivation of the CA1 of dorsal hippocampus on the CPA score and on conditioned context-induced increase in the expression of c-Fos in BLA in morphine withdrawn mice. **(A)** The experimental timeline and groups for the CPA procedure. **(B)** The influence of the inactivation of the CA1 of dorsal hippocampus on the CPA score in morphine withdrawn mice. Left panel: the typical injection site of muscimol in the CA1 of dorsal hippocampus. Scale bar = 500 μm. Right panel: the CPA score of each group (*n* = 10 in saline + saline + muscimol group, *n* = 7 in morphine + naloxone + muscimol group, *n* = 5 in morphine + naloxone + saline group, *p = 0.0003, compared with pre-test, two-way ANOVA, Bonferroni *post hoc* analysis). **(C)** The influence of the inactivation of the CA1 of dorsal hippocampus on conditioned context-induced increase in the expression of c-Fos in BLA in morphine withdrawn mice. Left panel: C-Fos positive neurons of the BLA in each group. Bar = 100 μm. Higher magnification images of boxed regions are shown on the left. Scale bar = 20 μm. Right panel: the average number of c-Fos positive neuron of the BLA in each group (*n* = 4 in saline + saline + muscimol group, *n* = 6 in morphine + naloxone + muscimol group, *n* = 4 in morphine + naloxone + saline group, *p = 0.001, one-way ANOVA following by Tukey *post hoc* analysis) Data are shown as the mean ± SEM.

To study the role of the CA1 of dorsal hippocampus in conditioned context-induced retrieval of morphine withdrawal memory, we examined the influence of the inactivation of the CA1 of dorsal hippocampus by the local injection of GABA_A_ receptor agonist muscimol on the CPA score. The mice were divided into three groups: saline + saline + muscimol group, morphine + naloxone + saline group and morphine + naloxone + muscimol group, and subjected to behavioral procedure as shown in Figure 2A. Left panel of Figure 2B showed a typical muscimol injection site in the CA1 of dorsal hippocampus. Right panel of Figure 2B showed the influence of the inactivation of the CA1 of dorsal hippocampus on the CPA score of post-test on 1 day after conditioning. The result showed that the inactivation of the CA1 of dorsal hippocampus by muscimol could abolish conditioned context-induced place aversion in morphine withdrawn mice on 1 day after conditioning (muscimol factor, *F*_(1, 38)_ = 13.75, *p* = 0.0007; test factor, *F*_(2, 38)_ = 5.357, *p* = 0.0089; muscimol × test, *F*_(2, 38)_ = 12.58, *p* < 0.0001; two-way ANOVA, Bonferroni *post hoc* analysis, right panel of Figure 2B). This result suggests that the CA1 of dorsal hippocampus plays an important role in conditioned context-induced retrieval of morphine-withdrawal memory.

### CA1 of Dorsal Hippocampus Is an Upstream Brain Region of the Activation of the BLA During Conditioned Context-Induced Retrieval of Morphine-Withdrawal Memory

To determine whether the CA1 of dorsal hippocampus is an upstream brain region of the activation of the BLA during the retrieval of morphine withdrawal memory, we examined the influence of the inactivation of the CA1 of dorsal hippocampus by the local injection of muscimol on the expression of c-Fos in the BLA. The behavioral procedure and the influence of the inactivation of the CA1 of dorsal hippocampus on CPA score of post-test on 1 day after conditioning were shown in Figures 2A,B. The mice were sacrificed at 90 min after post-test and c-Fos expression in the BLA was examined. Left panel of Figure 2C showed confocal images of c-Fos positive neurons and right panel of Figure 2C was the average number of c-Fos positive neurons in the BLA in each group. The result showed that the inactivation of the CA1 of dorsal hippocampus by muscimol could inhibit conditioned context-induced increase in the expression of c-Fos in the BLA on 1 day after conditioning (*F*_(2, 11)_ = 13.66, *p* = 0.0010; one-way ANOVA followed by Tukey’s multiple comparison test, right panel of Figure 2C). This result suggests that the CA1 of dorsal hippocampus is an upstream brain region of the activation of the BLA during conditioned context-induced retrieval of morphine-withdrawal memory.

### The Indirect Connection From CA1 of Dorsal Hippocampus to the BLA Is Enhanced in Mice With Conditioned Place Aversion

**FIGURE 3:**
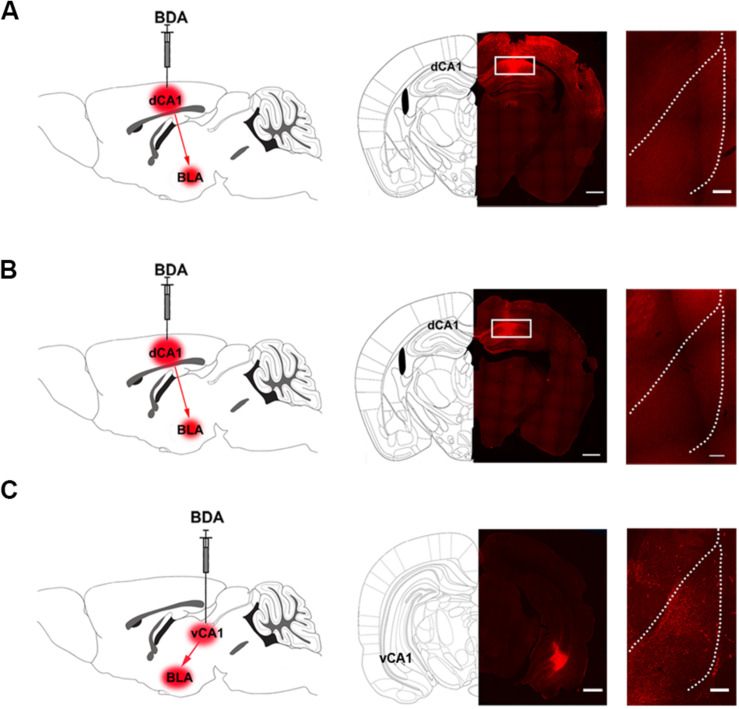
The projection from the CA1 of dorsal and ventral hippocampus to the BLA in mice. **(A)** Left panel: diagram of the injection site of BDA in the CA1 of dorsal hippocampus. Middle panel: the typical injection site of BDA (red-colored) in the dorsal CA1 of the hippocampus in mice of control group. Bar = 500 μm. Right panel: the BDA-positive fibers (red-colored) in the BLA from the CA1 of dorsal hippocampus on seventh day after BDA injection. Bar = 100 μm. **(B)** Left panel: diagram of the injection site of BDA in the CA1 of dorsal hippocampus. Middle panel: the typical injection site of BDA (red-colored) in the CA1 of dorsal hippocampus in mice with conditioned place aversion. Bar = 500 μm. Right panel: the BDA-positive fibers (red-colored) in the BLA from the CA1 of dorsal hippocampus on seventh day after BDA injection. Bar = 100 μm. **(C)** Left panel: diagram of the injection site of BDA in the CA1 of ventral hippocampus. Middle panel: the typical injection site of BDA (red-colored) in the CA1 of ventral hippocampus in mice of control group. Bar = 500 μm. Right panel: the BDA-positive fibers (red-colored) in the BLA from the CA1 of ventral hippocampus on seventh day after BDA injection. Bar = 100 μm.

We studied the pathway that mediated the CA1 of dorsal hippocampus-induced activation of the BLA during conditioned context-induced withdrawal memory retrieval. We examined the connection manner from the CA1 of dorsal hippocampus to the BLA. There are two possible connection manner from the CA1 of dorsal hippocampus to the BLA: one is the direct projection manner and another is the indirect manner. To examine whether there was a direct projection from the CA1 of dorsal hippocampus to the BLA, we locally injected anterograde tract tracer BDA into the CA1 of dorsal hippocampus of the mice in control group to anterograde label the axon terminals of projection neurons of the CA1 of dorsal hippocampus in the BLA. Middle panel of Figure 3A showed typical injection site of BDA in the CA1 of dorsal hippocampus. Right panel of Figure 3A showed the BDA-labeled terminals in the BLA at the seventh day after the injection. We could see that there were few BDA-labeled terminals in the BLA. This result suggests that the CA1 of dorsal hippocampus has few projections to the BLA. We also examined whether there was a change in the direct projection from the CA1 of dorsal hippocampus to the BLA in mice with CPA. The result showed that the CA1 of dorsal hippocampus still had few projections to the BLA in mice with CPA (Figure 3B). However, using a similar approach, our result showed that the CA1 of ventral hippocampus had a dense projection to the BLA (Figure 3C). This result was consistent with those of previous reports (Cenquizca and Swanson, 2007; Ciocchi et al., 2015; Kim and Cho, 2017).

**FIGURE 4:**
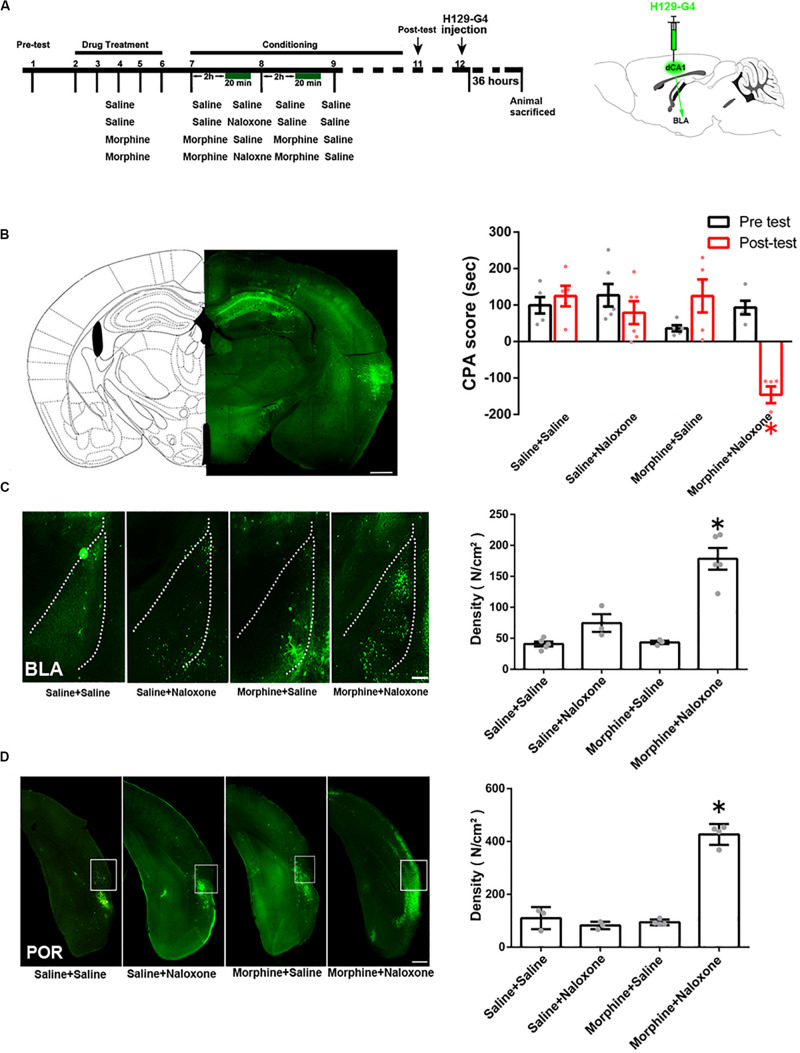
The indirect connection from the CA1 of dorsal hippocampus to the BLA in mice of control group and the change in the indirect projection from the CA1 of dorsal hippocampus to the BLA in mice with conditioned place aversion. **(A)** Left panel: the experimental timeline and groups for the CPA procedure. Right panel:diagram of the injection site of H129-G4 in the CA1 of dorsal hippocampus. **(B)** Left panel: the typical injection site of H129-G4 (green-colored) in the CA1 of dorsal hippocampus. Scale bar = 500 μm. Right panel: the CPA score of each group (*n* = 5 in saline + saline group, morphine + saline group and morphine + naloxone group, *n* = 6 in saline + naloxone group, *p < 0.0001, compared with pre-test, two-way ANOVA, Bonferroni *post hoc* analysis). **(C)** Left panel: H129-G4 positive neurons (green-colored) in the BLA in each group. Scale bar = 100 μm. Right panel: the density of H129-G4 positive neurons in the BLA in each group (*n* = 5 in saline + saline group and morphine + naloxone group, *n* = 3 in saline + naloxone and morphine + saline groups, *p < 0.0001, compare to saline + saline group, saline + naloxone group and morphine + saline group, one-way ANOVA following by Tukey *post hoc* analysis). **(D)** Left panel: H129-G4 positive neurons (green-colored) in the POR in each group. Scale bar = 100 μm. Right panel: the density of H129-G4 positive neurons in the POR in each group (*n* = 3 in saline + saline group and saline + naloxone group, *n* = 4 in morphine + saline group and morphine + naloxone group, *p < 0.0001, compare to saline + saline group, saline + naloxone group and morphine + saline group, one-way ANOVA following by Tukey *post hoc* analysis). Data are shown as the mean ± SEM.

**FIGURE 5:**
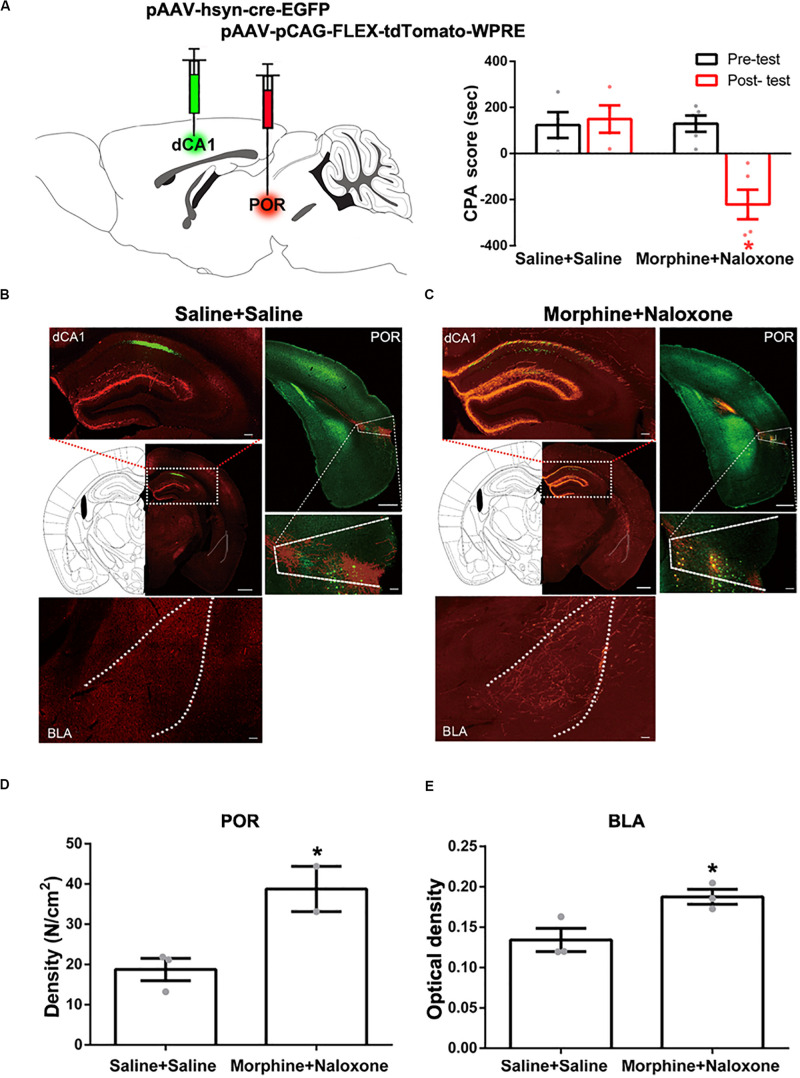
Neural circuit mediated by the POR from the CA1 of dorsal hippocampus to the BLA in saline-treated mice and mice with conditioned place aversion. **(A)** Left panel: diagram of the injection sites of viruses in the CA1 of dorsal hippocampus and the POR. Right panel: the CPA scores of saline + saline group and morphine + naloxone group (*n* = 4 in saline + saline group, *n* = 5 in morphine + naloxone group, *p = 0.004, compared with pre-test, two-way ANOVA, Bonferroni *post hoc* analysis). **(B)** Left top panel: the expression of Cre-EGFP (green-colored) in the CA1 of dorsal hippocampus after post-test in saline + saline group. Scale bar = 500 μm. The magnified image of the CA1 of dorsal hippocampus is shown on the top. Scale bar = 100 μm. Right top panel: Cre positive neurons (green-colored) and the expression of tdTomato-WPRE (red-colored) in the POR after post-test in saline + saline group. Scale bar = 500 μm. The magnified image of the POR is shown on the bottom. Scale bar = 100 μm. Down panel: the tdTomato positive fibers (red-colored) in the BLA in saline + saline group. Scale bar = 100 μm. **(C)** Left top panel: the expression of Cre-EGFP (green-colored) in the CA1 of dorsal hippocampus after post-test in morphine + naloxone group. Scale bar = 500 μm. The magnified image of the CA1 of dorsal hippocampus is shown on the top. Scale bar = 100 μm. Right top panel: Cre positive neurons (green-colored) and the expression of tdTomato-WPRE (red-colored) in the POR after post-test in morphine + naloxone group. Scale bar = 500 μm. The magnified image of the POR is shown on the bottom. Scale bar = 100 μm. Down panel: the tdTomato positive fibers (red-colored) in the BLA in morphine + naloxone group. Scale bar = 100 μm. **(D)** The density of Cre positive neurons of the POR in each group (*n* = 3 in saline + saline group, *n* = 2 in morphine + naloxone group, *p = 0.0360, compared to saline + saline group, student’s *t*-test). **(E)** The density of tdTomato positive fibers in the BLA in each group (*n* = 3 in each group, *p = 0.0352, compared to saline + saline group, student’s *t*-test). Data are shown as the mean ± SEM.

To study the indirect pathways from the CA1 of dorsal hippocampus to the BLA, we used H129-G4, a virus that could transmit through multiple synapses (Zeng et al., 2017), to examine whether this virus was able to reach the BLA. Mice were divided into four groups: saline + saline group, saline + naloxone group, morphine + saline group and morphine + naloxone group, as described in the method section and were subjected to behavioral paradigms as shown in left panel of Figure 4A. The injection site of H129-G4 in the CA1 of dorsal hippocampus (left panel) and behavioral results (right panel) were shown in Figure 4B (drug factor, *F*_(1, 34)_ = 9.830, *p* < 0.0001; test factor, *F*_(1, 34)_ = 4.608, *p* = 0.0039; drug × test, *F*_(3, 34)_ = 11.94, *p* < 0.0001; two-way ANOVA, Bonferroni *post hoc* analysis). Left panel of Figure 4C showed the confocal images of H129-G4 expression in the BLA and right panel of Figure 4C showed the density of H129-G4 positive neurons in each group. Result showed that the BLA had H129-G4 expression in saline + saline group and moreover the number of H129-G4 positive neurons significantly increased in morphine + naloxone group (*F*_(3, 12)_ = 30.34, *p* < 0.0001; one-way ANOVA followed by Tukey’s multiple comparison test), but H129-G4 had no significant changes in the saline + naloxone group and morphine + saline group (*F*_(__2_,_6__)_ = 5.114, *p* = 0.0506, one-way ANOVA followed by Tukey’s multiple comparison test). Left panel of Figure 4D showed the confocal images of H129-G4 expression in the POR and right panel of Figure 4D showed the density of H129-G4 positive neurons in each group. Result showed that the POR had H129-G4 expression in saline + saline group and the number of H129-G4 positive neurons significantly increased in morphine + naloxone group (*F*_(3, 10)_ = 118.2, *p* < 0.0001; one-way ANOVA followed by Tukey’s multiple comparison test), but H129-G4 had no significant changes in saline + naloxone group and morphine + saline group (*F*_(2, 7)_ = 0.8702, *p* = 0.4597, one-way ANOVA followed by Tukey’s multiple comparison test). These results suggest that there is an indirect pathway from the CA1 of dorsal hippocampus to the BLA. Moreover, it appears that the connection of this indirect pathway is enhanced in mice with CPA. To confirm this statement, we traced neural circuit mediated by the POR from the CA1 of dorsal hippocampus to the BLA using the two-step virus injection approach (Zingg et al., 2017). Left panel of Figure 5A showed the diagram of the virus injection site. Right panel of Figure 5A showed the CPA score of saline + saline group and morphine + naloxone group. The mice in these two groups were sacrificed and the virus expression was examined after post-test. The result showed that (1) after the injection of pAAV-hSyn-Cre-EGFP virus into the CA1 of dorsal hippocampus, the CA1 neurons of dorsal hippocampus were infected and labeled with EGFP protein (green-colored, left top panels of Figures 5B,C); (2) after the injection of pAAV-pCAG-FLEX-tdTomato-WPRE virus into the POR, POR neurons could be labeled with tdTomato protein (red-colored, right panels of Figures 5B,C), which suggested that those neurons were infected by pAAV-hSyn-Cre-EGFP virus from CA1 neurons of dorsal hippocampus; (3) axonal terminals of POR tdTomato positive neurons in the BLA could be labeled with tdTomato protein, which transported along axons of POR tdTomato positive neurons into the BLA (red-colored, down panels of Figures 5B,C). (4) Compared with the saline + saline group, the number of Cre positive neurons in the POR significantly increased in morphine + naloxone group (*t*_3_ = 3.628, *p* = 0.0360; student’s *t-*test, Figure 5D) and the density of tdTomato positive fiber in the BLA also significantly increased in the morphine + naloxone group (*t*_4_ = 3.129, *p* = 0.0352; student’s *t-*test, Figure 5E). This result confirms that the connection from the CA1 of dorsal hippocampus to the BLA through the POR indeed is enhanced in mice with CPA.

### The POR Is a Brain Region That Connects the CA1 of Dorsal Hippocampus to the Activation of the BLA in Conditioned Context-Induced Retrieval of Morphine-Withdrawal Memory

**FIGURE 6:**
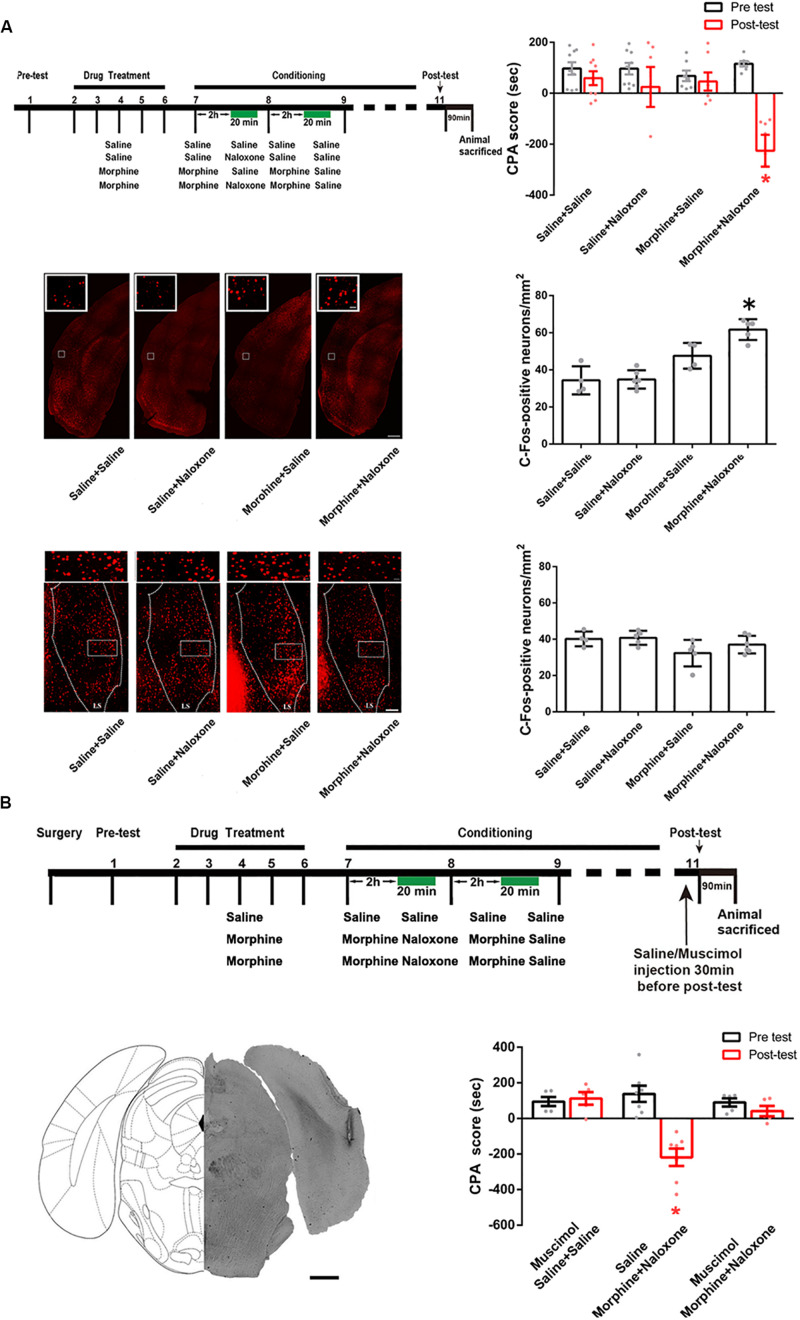
The influence of conditioned context on the expression of c-Fos in the POR and the LS in morphine withdrawn mice and the influence of the inactivation of the POR inactivation on the CPA score in morphine withdrawn mice. **(A)** The influence of conditioned context on the expression of c-Fos in the POR and the LS in morphine withdrawn mice. Left top panel: the experimental timeline and groups for the CPA procedure. Right top panel: the CPA score of each group (*n* = 9 in saline + saline group and saline + naloxone group, *n* = 7 in morphine + saline group and morphine + naloxone group *p = 0.001, compared with pre-test, two-way ANOVA, Bonferroni *post hoc* analysis). Left middle panel: C-Fos positive neurons in the POR, scale bar = 100 μm. Higher magnification images of boxed regions are shown on the top, scale bar = 20 μm. Right middle panel: average number of c-Fos positive neurons of the POR in each group (*n* = 4 in saline + saline and morphine + saline groups, *n* = 6 in saline + naloxone group, *n* = 5 in morphine + naloxone group, *p < 0.0001, one-way ANOVA following by Tukey *post hoc* analysis). Left down panel: C-Fos positive neurons in the LS, scale bar = 100 μm. Higher magnification images of boxed regions are shown on the top, scale bar = 20 μm. Right down panel: average number of c-Fos positive neurons of the LS in each group (*n* = 4 in saline + saline group, *n* = 5 in morphine + saline group and morphine + naloxone, *n* = 6 in saline + naloxone group, *p* = 0.0861, one-way ANOVA following by Tukey *post hoc* analysis). **(B)** The influence of the inactivation of the POR on CPA score in morphine withdrawn mice. Top panel: the experimental timeline and groups for the CPA procedure. Left down panel: the typical injection site of muscimol in the POR. Scale bar = 500 μm. Right down panel: the CPA score of each group (*n* = 5 in saline + saline + muscimol group, *n* = 5 in morphine + naloxone + muscimol group, *n* = 7 in morphine + naloxone + saline group, *p = 0.0019, compared with pre-test, two-way ANOVA, Bonferroni *post hoc* analysis). Data are shown as the mean ± SEM.

We examined possible downstream brain regions that connected the CA1 of dorsal hippocampus to the activation of the BLA in conditioned context-induced retrieval of morphine withdrawal memory. It has been known that the CA1 of dorsal hippocampus has a direct projection to the postrhinal cortex (POR) (Furtak et al., 2007) and the lateral septal nucleus (LS) (van Groen and Wyss, 1990). To study whether these two brain regions were downstream brain regions that connected the CA1 of dorsal hippocampus to the activation of the BLA in conditioned context-induced retrieval of morphine withdrawal memory, we examined whether conditioned context could activate the POR and the LS using c-Fos as a neuronal activation marker. Mice were randomly divided into four groups: saline + saline group, saline + naloxone group, morphine + saline group and morphine + naloxone group and were subjected to behavioral procedure as shown in left top panel of Figure 6A. The result showed that the mice in the morphine + naloxone group exhibited a strong aversion to withdrawal-paired compartment and thus spent less time in the withdrawal-paired compartment during the post-test than that during the pre-test, producing an increase in CPA score (drug factor, *F*_(1, 56)_ = 15.20, *p* = 0.0003; test factor, *F*_(3, 56)_ = 3.772, *p* = 0.0155; drug × test, *F*_(3, 56)_ = 5.597, *p* = 0.0020; two-way ANOVA, Bonferroni *post hoc* analysis, right top panel of Figure 6A), whereas mice in other groups did not exhibit a significant aversion to either compartment(drug factor, *F*_(1, 44)_ = 1.633, *p* = 0.2080; test factor, *F*_(2, 44)_ = 0.1456, *p* = 0.8649; drug × test, *F*_(2, 44)_ = 0.1785, *p* = 0.8371; two-way ANOVA, Bonferroni *post hoc* analysis, right top panel of Figure 6A). On this basis, we examined the expressions of c-Fos in the POR and the LS in each group. The result showed that the number of c-Fos positive neurons in the POR significantly increased in the morphine + naloxone group after re-exposure to conditioned context (*F*_(3, 15)_ = 22.20, *p* < 0.0001; one-way ANOVA followed by Tukey’s multiple comparison test, middle panels of Figure 6A), but did not in the LS (*F*_(3, 16)_ = 2.624, *p* = 0.0861; one-way ANOVA followed by Tukey’s multiple comparison test, down panels of Figure 6A). This result suggests that conditioned context can activate the POR. Then, we studied the role of the activated POR in the retrieval of morphine withdrawal memory by examining the influence of the inactivation of the POR by the intra-POR injection of muscimol on the CPA score. The mice were divided into three groups: saline + saline + muscimol group, morphine + naloxone + saline group and morphine + naloxone + muscimol group and were subjected to behavioral paradigms as shown in top panel of Figure 6B. Left down panel of Figure 6B showed a typical injection site in the POR and muscimol or saline was microinjected into the POR at 30 min before post-test. Right down panel of Figure 6B showed the average CPA scores of post-test and pre-test in each group. The mice in the morphine + naloxone + saline group exhibited a strong aversion to withdrawal-paired compartment, producing an increase in CPA score, whereas the mice in the saline + saline + muscimol group and the morphine + naloxone + muscimol group did not exhibit a significant aversion to either compartment (muscimol factor, *F*_(1, 28)_ = 15.32, *p* = 0.0005; test factor, *F*_(2, 28)_ = 7.493, *p* = 0.0025; muscimol × test, *F*_(2, 28)_ = 13.55, *p* < 0.0001; two-way ANOVA, Bonferroni *post hoc* analysis, right down panel of Figure 5B). This result suggests that the activation of the POR plays an important role in the retrieval of morphine withdrawal memory.

**FIGURE 7:**
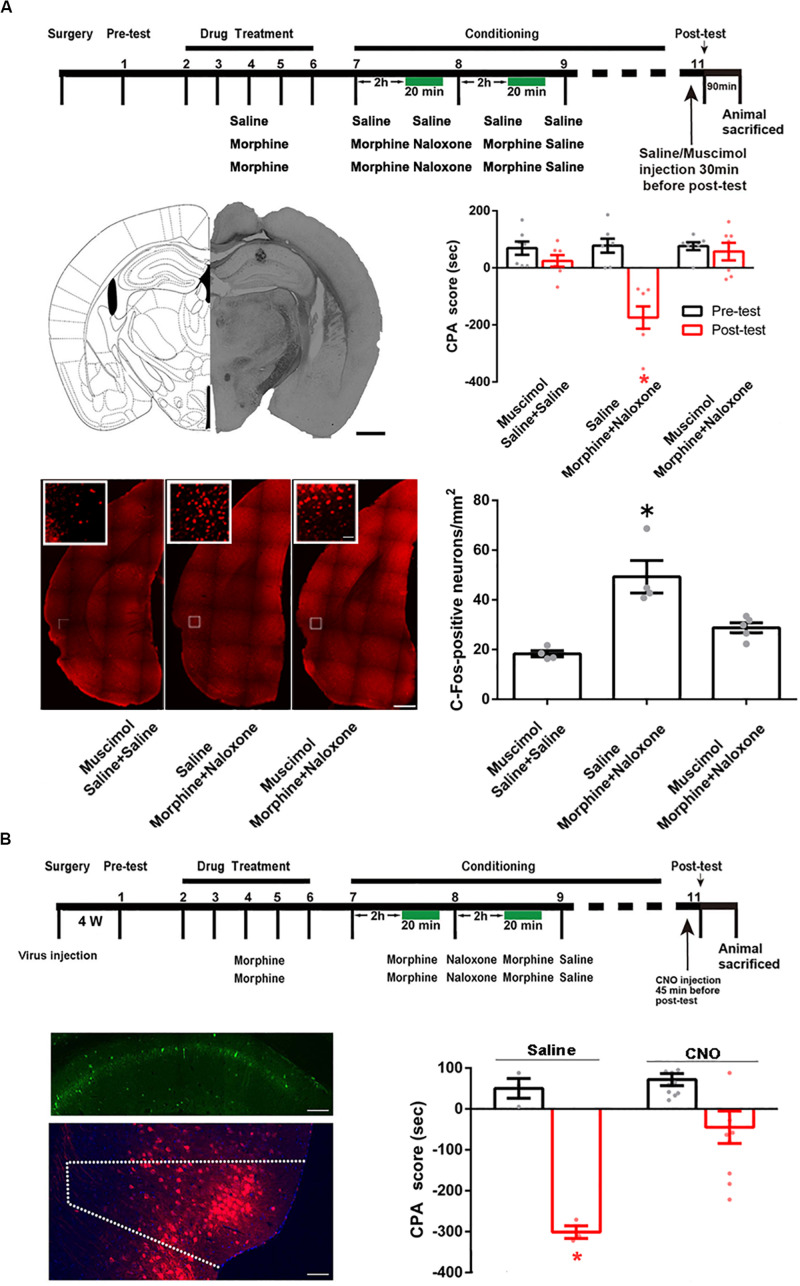
The influence of the inactivation of the CA1 of dorsal hippocampus on the increased expression of the c-Fos in the POR and the influence of chemical-genetic inactivation of the projection neurons from the CA1 of dorsal hippocampus to the POR on CPA score in morphine withdrawn mice. **(A)** The influence of the inactivation of the CA1 of dorsal hippocampus on the increased expression of the c-Fos in the POR. Top panel: the experimental timeline and groups for the CPA procedure. Left middle panel: the typical injection site of muscimol in the CA1 of dorsal hippocampus. Scale bar = 500 μm. Right middle panel: the CPA score of each group (*n* = 7 in each group, *p = 0.0004, compared with pre-test, two-way ANOVA, Bonferroni *post hoc* analysis). Left down panel: C-Fos positive neurons in the POR in each group. Scale bar = 100 μm. Higher magnification images of boxed regions are shown on the top. Scale bar = 20 μm. Right down panel: the average numbers of c-Fos positive neurons in the POR in each group (*n* = 4 in saline + saline + muscimol group and morphine + naloxone + saline group, *n* = 5 in morphine + naloxone + muscimol group, *p = 0.0007, one-way ANOVA following by Tukey *post hoc* analysis). **(B)** The influence of chemical-genetic inactivation of the projection neurons from the CA1 of dorsal hippocampus to the POR on CPA score in morphine withdrawn mice. Top panel: the CNO inhibit experimental timeline and groups for the CPA procedure. Left middle panel: expression of hM4Di (Gi) (green-colored) in the CA1 of dorsal hippocampus 4 weeks after the virus injection. Scale bar = 100 μm. Left down panel: expression of WGA-Cre (red-colored) in the POR 4 weeks after the virus injection. Scale bar = 100 μm. Right down panel: the CPA scores of saline group and CNO group (*n* = 10 in CNO group, *n* = 3 in saline group, *p = 0.0083, compared with pre-test, two-way ANOVA, Bonferroni *post hoc* analysis). Data are shown as the mean ± SEM.

We studied whether the CA1 of dorsal hippocampus was an upstream brain region of the activation of the POR during conditioned context-induced retrieval of morphine withdrawal memory by examining the influence of the inactivation of the CA1 of dorsal hippocampus on the increased expression of the c-Fos in the POR by the intra-dorsal CA1 injection of GABA_A_ agonist muscimol. The mice were divided into three groups: saline + saline + muscimol group, morphine + naloxone + saline group and the morphine + naloxone + muscimol group, as described in the method section and were subjected to behavioral procedure as shown in top panel of Figure 7A. Muscimol or saline was microinjected into the CA1 of dorsal hippocampus at 30 min before post-test. Left middle panel of Figure 7A showed a typical injection site in the CA1 of dorsal hippocampus. Right middle panel of Figure 7A showed that the average CPA score of post-test in the morphine + naloxone + saline group was significantly different from that of pre-test, but in the saline + saline + muscimol group and the morphine + naloxone + muscimol group, the average CPA score of post-test was not significantly different from that of pre-test (muscimol factor, *F*_(1, 36)_ = 23.61, *p* < 0.0001; test factor, *F*_(2, 36)_ = 10.74, *p* = 0.0002; muscimol × test, *F*_(2, 36)_ = 11.60, *p* = 0.0001; two-way ANOVA, Bonferroni *post hoc* analysis). Then, the mice were sacrificed at 90 min after post-test and the c-Fos positive neurons in the POR were examined (left down panel of Figure 7A). The average number of c-Fos positive neurons in the POR in the morphine + naloxone + muscimol group significantly decreased than that of the morphine + naloxone + saline group (*F*_(2, 10)_ = 16.43, *p* = 0.0007; one-way ANOVA followed by Tukey’s multiple comparison test, right down panel of Figure 7A), suggesting that the inactivation of the CA1 of dorsal hippocampus by muscimol significantly suppressed the increased expression of c-Fos in the POR by conditioned context. This result suggests that the CA1 of dorsal hippocampus of the hippocampus is an upstream brain region of the activation of the POR during conditioned context-induced retrieval of morphine withdrawal memory. We also studied the role of the projection neurons from the CA1 of dorsal hippocampus to the POR in conditioned context-induced retrieval of morphine withdrawal memory by examining the influence of chemical-genetic inactivation of the projection neurons from the CA1 of dorsal hippocampus to the POR on CPA score. AAV-hSyn-DIO-hM4D(Gi)-EGFP was stereotaxically injected into the CA1 of dorsal hippocampus and AAV-hSyn-mCherry-IRES-WGA-Cre was injected into the POR of mice. Four weeks after the virus injection, the expression of hM4D(Gi)-EGFP in the CA1 of dorsal hippocampus (top panel) and WGA-Cre in the POR (down panel) were examined (left down panel of Figure 7B). The mice were divided into two groups: saline group and clozapine-n-oxide (CNO) group and were treated with saline or CNO (i.*p*.) at 45 min before post-test. Results showed that the inhibition of projection neurons from the CA1 of dorsal hippocampus to the POR by CNO could significantly decrease the CPA score (CNO factor, *F*_(1, 22)_ = 33.44, *p* < 0.0001; test factor, *F*_(1, 22)_ = 11.76, *p* = 0.0024; CNO × test, *F*_(1, 22)_ = 8.416, *p* = 0.0083; two-way ANOVA, Bonferroni *post hoc* analysis, right down panel of Figure 7B). This result suggests that the projection neurons from the CA1 of dorsal hippocampus to the POR play an important role in the retrieval of morphine-withdrawal memory.

**FIGURE 8:**
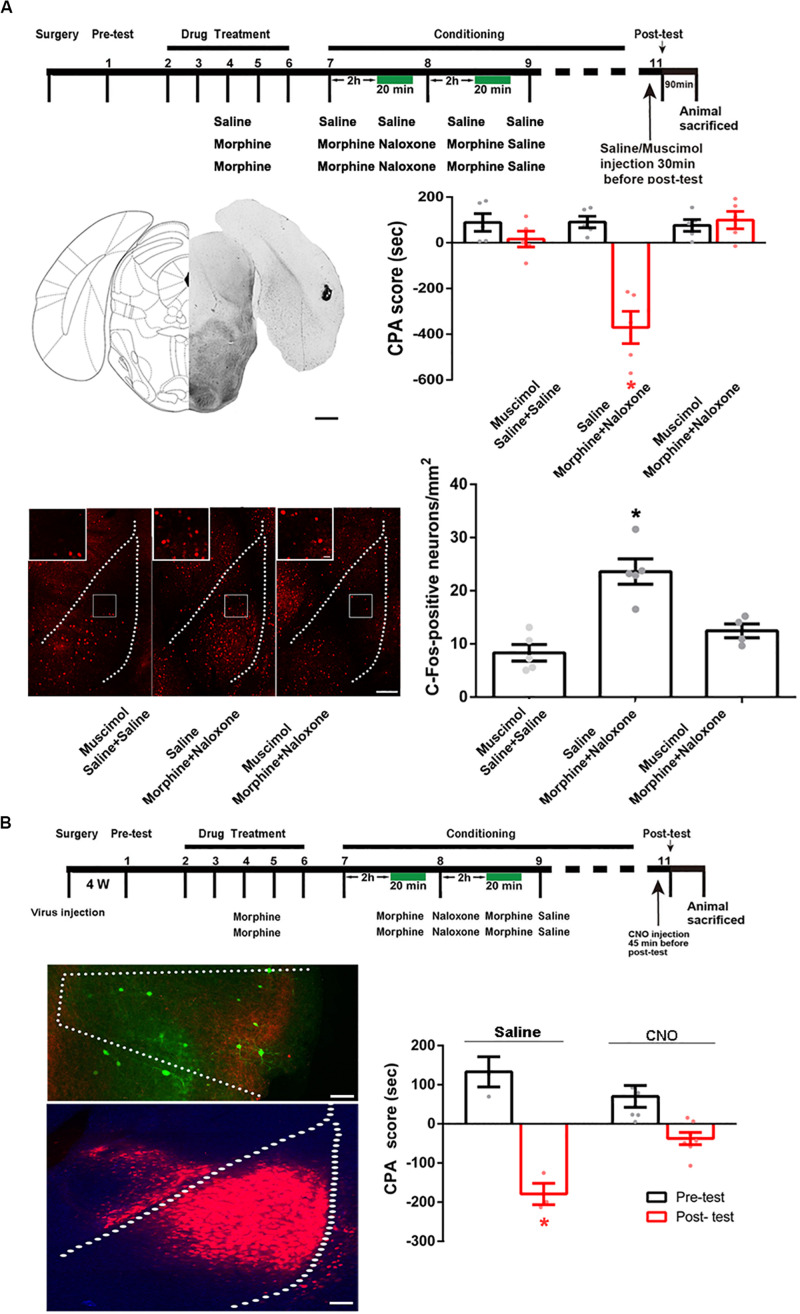
The influence of inactivation of the POR on the increased expression of c-Fos in the BLA and the influence of chemical-genetic inactivation of the projection neurons from the POR to the BLA on CPA score in morphine withdrawn mice. **(A)** The influence of the inactivation of the POR on the increased expression of c-Fos in the BLA. Top panel: experimental timeline and groups for the CPA procedure. Left middle panel: the typical injection site of muscimol in the POR. Scale bar = 500 μm. Right middle panel: the CPA score of each group (*n* = 5 in each group, *p < 0.0001, compared with pre-test, two-way ANOVA, Bonferroni *post hoc* analysis). Left down panel: C-Fos positive neurons in the BLA in each group. Scale bar = 100 μm. Higher magnification images of boxed regions are shown on the top. Scale bar = 20 μm. Right down panel: the average numbers of c-Fos positive neurons in the BLA in each group (*n* = 5 in saline + saline + muscimol group and morphine + naloxone + saline group, *n* = 4 in morphine + naloxone + muscimol group, *p = 0.0003, one-way ANOVA following by Tukey *post hoc* analysis). **(B)** The influence of chemical-genetic inactivation of the projection neurons from the POR to the BLA on CPA score in morphine withdrawn mice. Top panel: the CNO inhibit experimental timeline and groups for the CPA procedure. Left middle panel: expression of hM4Di (Gi) (green-colored) in the POR 4 weeks after virus injection. Scale bar = 100 μm. Left down panel: expression of WGA-Cre (red-colored) in the BLA 4 weeks after virus injection. Scale bar = 100 μm. Right down panel: the CPA scores of saline group and CNO group (*n* = 7 in CNO group, *n* = 4 in saline group, *p = 0.0009, compared with pre-test, two-way ANOVA, Bonferroni *post hoc* analysis). Data are shown as the mean ± SEM.

Then, we studied whether the BLA was a downstream brain region activated by the POR during conditioned context-induced retrieval of morphine withdrawal memory by examining the influence of the inactivation of the POR on the increased expression of the c-Fos in the BLA by the intra-POR injection of GABA_A_ agonist muscimol. The mice were divided into three groups: saline + saline + muscimol group, morphine + naloxone + saline group and morphine + naloxone + muscimol group, as described in the method section and were subjected to behavioral paradigms as shown in top panel of Figure 8A. Muscimol or saline was microinjected into the POR at 30 min before post-test. Left middle panel of Figure 8A showed a typical injection site in the POR. Right middle panel of Figure 8A showed that the average CPA score of post-test in the morphine + naloxone + saline group was significantly different from that of pre-test, but in the saline + saline + muscimol group and the morphine + naloxone + muscimol group, the average CPA score of post-test were not significantly different from that of pre-test (muscimol factor, *F*_(1, 24)_ = 25.10, *p* < 0.0001; test factor, *F*_(2, 24)_ = 17.31, *p* < 0.0001; muscimol × test, *F*_(2, 24)_ = 19.03, *p* < 0.0001; two-way ANOVA, Bonferroni *post hoc* analysis). Then, the mice were sacrificed at 90 min after post-test and the c-Fos positive neurons in the BLA were examined (left down panel of Figure 8A). The average number of c-Fos positive neurons in the BLA in the morphine + naloxone + muscimol group was lower than that of the morphine + naloxone + saline group (*F*_(2, 11)_ = 18.69, *p* = 0.0003; one-way ANOVA followed by Tukey’s multiple comparison test, right down panel of Figure 8A), suggesting that the inactivation of the POR by muscimol significantly suppressed the increased expression of c-Fos in the BLA by conditioned context. This result suggests that BLA is a downstream brain region activated by the POR during conditioned context-induced retrieval of morphine withdrawal memory. We also studied the role of the projection neurons from the POR to the BLA in conditioned context-induced retrieval of morphine-withdrawal memory by examining the influence of chemical-genetic inactivation of the projection neurons from the POR to the BLA on CPA score. AAV-hSyn-DIO-hM4D(Gi)-EGFP was stereotaxically injected into the POR and AAV-hSyn-mCherry-IRES-WGA-Cre was injected into the BLA of mice. Four weeks after the virus injection, the expression of hM4D(Gi)-EGFP in the POR (top panel) and WGA-Cre in BLA (down panel) were examined in left down panel of Figure 8B. The mice were divided into two groups: saline group and CNO group and treated with saline or CNO (i.p.) at 45 min before post-test. Results showed the inhibition of projection neurons from the POR to the BLA by CNO could significantly decrease the CPA score (CNO factor, *F*_(1, 18)_ = 5.008, *p* = 0.0381; test factor, *F*_(1, 18)_ = 62.07, *p* < 0.0001; CNO × test, *F*_(1, 18)_ = 15.60, *p* = 0.0009; two-way ANOVA, Bonferroni *post hoc* analysis, right down panel of Figure 8B). This result suggests that the projection neurons from the POR to the BLA play an important role in the retrieval of morphine withdrawal memory.

## Discussion

Considerable evidence suggests that both the dorsal and ventral hippocampus are important for conditioned context-induced retrieval of fear memory (Phillips and LeDoux, 1992; Holt and Maren, 1999; Hobin et al., 2006). A novel finding of the present study was that for the retrieval of morphine withdrawal memory, only the dorsal hippocampus, but not the ventral hippocampus, played an important role.

The downstream connections of the dorsal hippocampus have been studied widely. The CA1 area, as the major output structure of the dorsal hippocampus (Daumas et al., 2005), sends its primary direct projections to the POR, the entorhinal cortex, the lateral septal nucleus, the dorsal subiculum (Roy et al., 2017), the caudal part of the lateral septal nucleus, the medial and lateral mammillary nuclei and the anterior thalamic complex (Fanselow and Dong, 2010) and then, through sequential, multisynaptic, and presumably feed-forward excitatory approaches, sends indirect projections to its downstream cortical and subcortical brain regions (Gaykema et al., 1991; Jay and Witter, 1991; Cenquizca and Swanson, 2006; Cenquizca and Swanson, 2007; Cadiz-Moretti et al., 2016). However, no reports showed that the BLA was a downstream direct projection region of the CA1 of dorsal hippocampus. This is consistent with our result that the CA1 of the dorsal hippocampus has no a direct projection to the BLA.

To study the indirect connection from the CA1 of dorsal hippocampus to the BLA, we used the H129-G4 transneuronal tracing technique reported by Zeng et al. (2017). H129-G4 was obtained by inserting binary tandemly connected GFP cassettes into the genomes of the Herpes simplex virus type 1 strain 129 (H129) (Zeng et al., 2017) and was a newly developed anterograde neuronal circuit tracing tool. H129-G4 is capable of transmitting through multiple synapses, labeling the neurons by green florescent protein and visualizing the morphological details of the labeled neurons (Zeng et al., 2017). Using H129-G4, our result showed that there was an indirect connection from the CA1 of dorsal hippocampus to the BLA and this indirect connection appeared to be significantly enhanced in mice with CPA because reached amount of H129-G4 from the CA1 of dorsal hippocampus to the BLA, in mice with CPA significantly increased. However, the neural substrates of the enhanced flow of H129-G4 in the indirect connection from the CA1 of dorsal hippocampus to the BLA in mice with CPA remain to be unknown. It may be related to increased synapses in the indirect connection from the CA1 of dorsal hippocampus to the BLA in mice with CPA.

We further studied what brain regions connect the CA1 of dorsal hippocampus to the BLA during conditioned context-induced retrieval of morphine withdrawal memory. The POR is bordered by the following structures: the caudal end of the angular bundle, the caudal part of the perirhinal cortex, the ventral temporal association area, and the dorsal part of the medial entorhinal cortex (Qi et al., 2019). The POR receives direct projections from the CA1 of dorsal hippocampus (Furtak et al., 2007; Agster and Burwell, 2013) and sends projections onto a number of cortical and subcortical areas (Delatour and Witter, 2002; Agster and Burwell, 2009). Among them, the POR has a dense projection to the BLA (Burgess et al., 2016). Therefore, it is possible that the CA1 of the dorsal hippocampus, the POR and the BLA constitute a series connection that participates in conditioned context-induced retrieval of morphine withdrawal memory. This hypothesis is confirmed by our results using trans-neuronal virus tracing technique combined with chemical-genetic method.

Previous studies showed that the POR was implicated in processing contextual and visuospatial information and contributed to perceptual processing of spatial and contextual environmental information (Bucci et al., 2000; Burwell et al., 2004). The role of the POR in coding of context and egocentric spatial relations has been attributed to its input to the hippocampus. The hippocampus receives spatial information from the POR via the medial entorhinal cortex and non-spatial information from the perirhinal cortex via the lateral entorhinal cortex and then binds these two streams of information to represent context (Heimer-McGinn et al., 2017). A novel finding of present study is that the activated hippocampus by environmental context activates the POR again and then induces the retrieval of morphine withdrawal memory by the activation of the BLA.

To summarize, our results suggest that a conditioning-strengthened indirect circuit from the CA1 of dorsal hippocampus to the BLA through the POR participates in the retrieval of morphine withdrawal memory. Interestingly, in a previous study by Alice H. Luo et al., they found that for reward response, this indirect pathway that linked context with reward was from the CA3 of dorsal hippocampus to the ventral tegmental area (VTA) that used the lateral septum (LS) as a relay (Luo et al., 2011). The future study will examine the neural substrates of the enhanced indirect connection from the CA1 of dorsal hippocampus to the BLA in mice with CPA.

## Data Availability Statement

The datasets generated for this study are available on request to the corresponding author.

## Ethics Statement

The animal study was reviewed and approved by the Animal Care and Use Committee of Shanghai Medical College of the Fudan University.

## Author Contributions

QM and YF designed and performed the experiments, analyzed the data, and drafted and revised the manuscript. ZC, DS, JS, HS, LY, DC, MC, FZ, and M-HL analyzed and interpreted the data. PZ and BL designed the experiments, analyzed the data, and drafted and revised the manuscript. All authors contributed to the article and approved the submitted version.

## Conflict of Interest

The authors declare that the research was conducted in the absence of any commercial or financial relationships that could be construed as a potential conflict of interest.
